# Therapeutic Potential of Fecal Microbiota Transplantation in Type 2 Diabetes Mellitus: A Systematic Review

**DOI:** 10.7759/cureus.70642

**Published:** 2024-10-01

**Authors:** Muhammad Rizwan Aslam, Alekya Perala, Annetta V Wishart, Ranim K Hamouda, Entesar Elsaady, Safeera Khan

**Affiliations:** 1 Internal Medicine, California Institute of Behavioral Neurosciences & Psychology, Fairfield, USA; 2 Research, California Institute of Behavioral Neurosciences & Psychology, Fairfield, USA; 3 Research, California Institute of Behavioral Neurosciences & Psychology, Fairfield, California, USA; 4 Internal Medicine/Hematology, California Institute of Behavioral Neurosciences & Psychology, Fairfield, USA

**Keywords:** fecal microbiota transplantation (fmt), insulin resistance, therapy, treatment, types 2 diabetes

## Abstract

Diabetes mellitus is a chronic metabolic disease characterized by insulin resistance and hyperglycemia. It can cause various complications, which result in significant morbidity and mortality. There are multiple treatment options available to combat this disease; however, despite this, the incidence of type 2 diabetes mellitus is continuously increasing. Some promising results have shown that dysbiosis has a role in the pathogenesis of type 2 diabetes mellitus and fecal microbiota transplantation (FMT) in animals; however, the usage of FMT in humans needs further clarification and review. We explored PubMed, Popline, and Cochrane Library to identify relevant papers. Eight articles were then finalized after screening and applying eligibility criteria. These articles explored the role of the therapeutic efficacy of FMT in insulin resistance and hyperglycemia. The studies showed that the FMT had a positive impact on managing hyperglycemia and insulin resistance, which is evident in the decline of blood glucose and HBA1c levels and the rise of insulin and C-peptides. In addition, FMT also helped to control other risk factors such as hyperlipidemia and blood pressure; however, the impact on weight loss is not convincing. FMT also influenced the levels of some microbiota, which could be involved in controlling hyperglycemia and insulin resistance. Due to limited control trials and study periods and the small sample size of diabetic patients, more research is needed to explore the impact of FMT in controlling type 2 diabetes mellitus.

## Introduction and background

Diabetes mellitus has now become a serious health hazard, reaching pandemic proportions. Around 529 million people were living with diabetes worldwide in 2021, with a prevalence rate of 6.1%, and by 2050, the number is expected to increase to 1.31 billion. Type 2 diabetes accounted for the majority of the cases, with an estimated 96% [1]. Diabetes is affecting the quantity and quality of life and also having a detrimental impact on health expenditure. In 2022, the USA spent around $412.9 billion on diabetes [2]. In the UK, the National Health Service (NHS) spends around £10 billion a year, which is around 10% of its budget [3].

Diabetes mellitus is a chronic metabolic disease characterized by hyperglycemia and insulin resistance, causing significant morbidity and mortality. It can cause complications such as diabetic nephropathy, neuropathy, and retinopathy, and there is also an increased risk of having cardiovascular diseases [4] and cancer [5]. Despite newer treatment modalities such as SGLT2 and GLP-1 agonists and other oral hypoglycemic agents, diabetes is still ineffectively controlled. In addition, antihyperglycemic drugs have adverse effects; therefore, innovative therapeutic approaches are needed to combat this pandemic.

Gut microbiota is the community of organisms comprising approximately 1,000 different species of bacteria, viruses, archaea, and unicellular eukaryotes [6]. Gut microbiota is involved in glucose metabolism and insulin sensitivity [7]. Studies have shown that alterations in Gut microbiota, which is known as dysbiosis, have a crucial role in the pathogenesis of type 2 diabetes [8] and its complications [9]. Fecal microbiota transplantation (FMT) is now emerging as a new therapeutic option for treating diabetes, and several studies in animals have shown improvement in hyperglycemia and diabetes progression [10-12].

However, there is still a knowledge gap in the usage of FMT in type 2 diabetic patients. In this systematic review, we aim to include clinical trials, observational studies, and case reports focusing on the role of FMT in type 2 diabetes mellitus and insulin resistance, as well as an overview of its efficacy.

## Review

Methodology

This systematic review followed the Preferred Reporting Items for Systematic Review and Meta-Analysis (PRISMA) 2020 guidelines [13].

Search Sources and Strategy

We explored PubMed, Cochrane, and Popline databases to search for the studies and relevant articles (Table 1). The included keywords were “fecal microbiota transplantation, gut microbiota, type 2 diabetes mellitus, treatment, therapy.” In PubMed, we used different combinations of these keywords, such as “fecal microbiota transplantation AND type 2 diabetes mellitus AND treatment” and “fecal microbiota transplantation AND type 2 diabetes mellitus AND therapy.”

**Table 1:** MeSH terms utilized and the number of studies identified from each database

Search Strategy	Database	Studies
Fecal microbiota transplantation AND type 2 diabetes mellitus AND treatment	PubMed	87
Fecal microbiota transplantation AND type 2 diabetes mellitus AND therapy	PubMed	77
("Fecal Microbiota Transplantation"[Mesh]) AND (( "Diabetes Mellitus, Type 2/metabolism"[Mesh] OR "Diabetes Mellitus, Type 2/microbiology"[Mesh] OR "Diabetes Mellitus, Type 2/therapy"[Mesh] ))	PubMed Mesh	40
Fecal microbiota transplantation AND type 2 diabetes mellitus AND treatment	Cochrane Library	11
Fecal microbiota transplantation AND type 2 diabetes mellitus AND treatment	Popline	79

Eligibility

The inclusion criteria consisted of articles written and published in English, articles from all geographical locations, and time period, and articles focusing on FMTs, type 2 diabetes mellitus, and Insulin resistance. Other inclusion criteria included articles on humans and all age groups and genders, study types, and designs. These inclusion criteria were considered to collect as much relevant data as possible for the systematic review. Articles that focused on other types of diabetes (except type 2 diabetes mellitus or insulin resistance), grey literature, articles in languages other than English, and animal studies were excluded.

Selection Process

Studies and articles from the above databases were transferred to Endnote, and duplicates were removed. Afterward, a screening process was initiated, and each study and article were assessed by title and abstract. Shortlisted studies and articles were then fully evaluated by reading the full text. Only papers that met the eligibility criteria were shortlisted. Discussions were held with co-authors in case of any concerns regarding shortlisting and eligibility and finalized by mutual agreement.

Quality Appraisal

After screening, studies, and articles were checked for quality using quality appraisal tools. Randomized controlled trials (RCTs) were evaluated with the Cochrane bias assessment tool (Table 2). Non-RCTs and observational studies were assessed using the Newcastle Ottawa tool (Table 3). In the narrative review and meta-analysis case, the Scale for Assessment of Narrative Review Article (SANRA) (Table 4) and AMSTAR checklists (Table 5) were used, respectively. Finally, the JB check tool (Table 6) was used for a case report. Studies and articles that fulfilled the quality appraisal tools were included in the systematic review.

**Table 2:** Cochrane bias assessment for randomized controlled trial

Bias	Kootte et al., 2017 [14]	Ng et al., 2022 [15]	Wu et al., 2023 [16] (study not included)
Random sequence generation (selection bias)	Low risk	Low risk	Low risk
Allocation concealment (selection bias)	Unclear	Unclear	Unclear
Blinding of participants and personnel (performance bias)	Low risk	Los Risk	Unclear
Blinding of outcome assessment (detection bias)	Unclear	Low Risk	Unclear
Incomplete outcome data (attrition bias)	Low risk	Some concerns	Low risk
Selective outcome bias (reporting bias)	Low risk	Low Risk	Low risk
Other bias	Some concerns	Some concerns	Some concerns
Overall	Low risk 64%	Low risk 71%	Some concerns 50%

**Table 3:** Newcastle Ottawa scale for non-randomized controlled trial and observational studies BMI - Body Mass Index

	Ding et al., 2022 [17]	Su et al., 2022 [18]	Wu et al., 2022 [19]
Is the case definition adequate	1	1	1
Representativeness of the cases	1	1	1
Selection of controls	1	1	1
Definition of controls	1	0	1
Comparability of cases and controls based on the design or analysis	1 (on sex, BMI, clinical parameters)	1	1
Ascertainment of exposure	-	1	1
The same method of ascertainment for cases and controls	1	1	1
Non-response rate	0	0	0
	75%	75%	87.5%

**Table 4:** Scale for Assessment of Narrative Review Articles (SANRA) checklist

SANRA checklist	Guzzardi et al., 2023 [20]
Justification	2
Statements of concrete aims or formulation of the question	2
Description of literature search	2
Referencing	2
Scientific reasoning	2
Appropriate presentation of data	2

**Table 5:** A Measurement to Assess Systematic Review (AMSTAR)

AMSTAR 2 criteria	Qiu et al., 2023 [21]
Population, intervention, comparison, and outcome (PICO) components	Yes
Pre-established review methods and any substantial protocol deviation	Yes
Justification for selection of study designs	Yes
Search strategy for the literature explained?	Yes
Duplicate study selection performed.	Yes
Duplicate data extraction was performed.	Yes
Justification for the excluded studies provided	Yes
Detailed description of the included studies	Yes
Assessment of the risk of bias (RoB) in individual studies	Yes
Reporting on the funding sources	No
Appropriate methods used for statistical combination of results	Yes
Impact of RoB in individual studies on the results of the meta-analysis	Yes
RoB used in interpreting the results	Yes
Explanation of heterogeneity in the results	Yes
Investigation of publication bias and its impact on the results?	Yes
Conflict of interest and funding	No

**Table 6:** Joanna Briggs Institute critical appraisal checklist for case reports

JBI check tool	Cai et al., 2018 [22]
Were the patient's demographic characteristics clearly described?	Yes
Was the patient’s history clearly described and presented as a timeline?	Yes
Was the current clinical condition of the patient on presentation clearly described?	Yes
Were diagnostic tests or assessment methods and the results clearly described?	Yes
Was the intervention(s) or treatment procedure(s) clearly described?	Yes
Was the post-intervention clinical condition clearly described?	Yes
Were adverse events (harms) or unanticipated events identified and described?	Insufficient
Does the case report provide takeaway lessons?	Yes

Data Extraction

After the above process, data was then extracted and recorded in the sheets. The extracted information was the study's title, author names, date of publication, the country where the study was conducted, number of participants/subjects, and results.

Results

Study Identification and Selection

**Figure 1:**
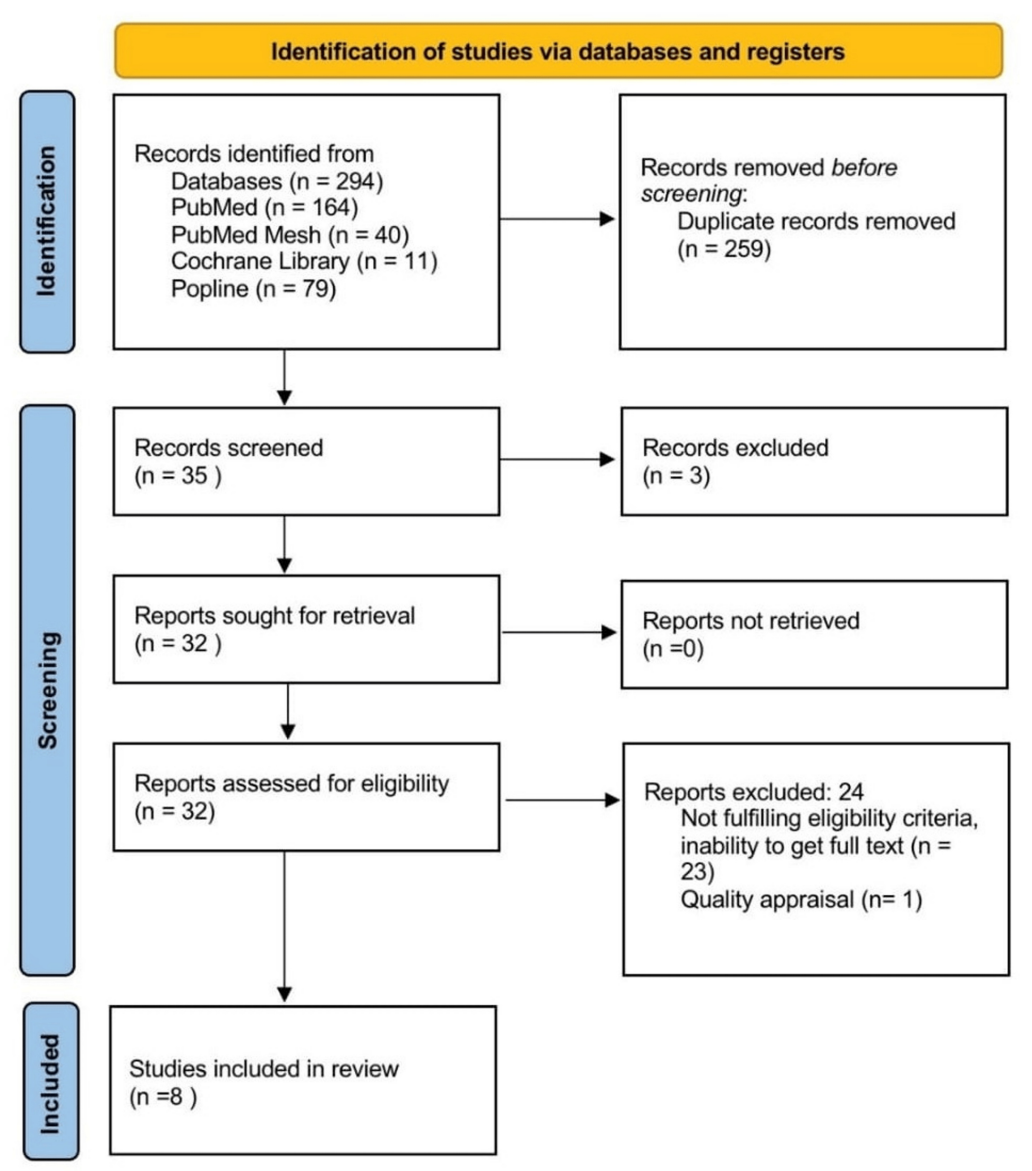
PRISMA flow diagram PRISMA: Preferred Reporting Items for Systematic Review [13]

We searched 294 articles using various combinations of keywords on relevant databases. After removing the duplicates, 35 articles remained. The screening was then conducted by reviewing titles and abstracts; 32 articles were shortlisted. These shortlisted articles were further assessed by going through full text for eligibility, inability to get the full text, and quality appraisal; eight articles were then finalized for systematic review. The selection method and process are shown in Figure 1.

Outcome Measured

Data extracted from the finalized articles were divided into primary and secondary outcomes. Primary outcomes include fasting blood sugar, postprandial glucose, HbA1c, insulin levels, Homeostatic Model Assessment for Insulin Resistance (HOMA-IR), and C-peptide. Lipid profile (LDL, HDL, cholesterol, and triglycerides (TG)), blood pressure, and weight loss were included in the secondary outcome. Some of the studies also assessed the type of microbiota involved in improving glycemic control, which is also included.

Characteristics of Studies

The eight final articles (Table 7) included consisted of different studies. Two were RCTs, one was a NRCT, and the other two were observational studies. One meta-analysis and one narrative review were also included. In addition to that, one case report was also added to our systematic review. A total of 785 participants were part of the finalized articles. All participants had FMT, and various glycemic control parameters, such as fasting blood glucose, postprandial glucose, HbA1c, and HOMA-IR, were assessed at baseline and after FMT. Some studies also evaluated the insulin, C peptide levels, lipid profile, and type of microbiome involved in glucose metabolism and improvement.

**Table 7:** Characteristics of the included studies RCT - Randomized Controlled Trial, NCT - Non-randomized Controlled Trial, FMT - Fecal Microbiota Transplantation, FBG - Fasting Blood Glucose, BP - Blood Pressure, WMT - Washed Microbiota Transplantation, TG - Triglycerides, LDL - Low-density Lipoprotein, BMI - Body Mass Index, T2DM - Type 2 Diabetes Mellitus, HOMA-IR - Homeostatic Model Assessment for Insulin Resistance, LSI - Life Style Intervention

Author and year of publication	Type of study	Intervention studied	No. of participants	Parameters assessed	Results	Conclusion
Kootte et al., 2017 [14]	RCT	Improvement of insulin sensitivity after fecal microbiota transplantation (FMT)	38 participants - 12 autologous and 26 allogenic	Glucose, HbA1c, Insulin, sensitivity, Lipid profile, weight	Allogenic fecal microbiota transplantation (FMT) improved insulin sensitivity and HbA1c after 6 weeks and increased fasting triglycerides (TG). No effect on glucose and weight loss.	Beneficial Intestinal microbiota can be used to identify individuals at risk of developing insulin resistance and treat them with targeted microbiota-based interventions.
Ng et al., 2022 [15]	RCT	Efficacy of fecal microbiota transplantation (FMT) with and without lifestyle intervention (LSI)	52 participants - 17 each in FMT and FMT plus LSI. 18 in sham plus LSI.	Fasting blood pressure (FBG), HbA1c, Insulin, lipid profile, weight	At week 24, there was no significant difference in fasting glucose level, fasting insulin level, HbA1c, and weight loss in each group with baseline, except lipid profile was improved in the FMT and LSI groups.	Repeated FMT resulted in a sustained increase in microbiota engraftment in type 2 obese diabetic patients.
Ding et al., 2022 [17]	NCT	Response of fecal microbiota transplantation (FMT) in T2 diabetic patients with regards to glycemic control	37 participants -17 Type 2 diabetic and 20 control	Fasting blood glucose (FBG), Postprandial glucose, HbA1c, C peptide, lipid profile, weight, BP	After 12 weeks, FMT had a positive effect on FBG, Postprandial glucose, HbA1c, C peptide, and lipid profile (decreased LDL and TG). No significant weight loss and BP reduction occurred in any group.	FMT is a potential treatment, however, it requires further broad clinical research with active FMT components.
Su et al., 2022 [18]	Observational Study	Assessment of response of fecal microbiota transplantation (FMT) and FMT plus diet in T2 diabetic patients	13 participants - 8 were included in the diet group and 5 in the diet plus FMT group	Fasting blood glucose (FBG), HbA1c, lipid profile, blood pressure (BP), weight	On days 20 and 90, there was an improvement of parameters in both groups but better in the FMT group.	FMT has beneficial effects on glycemic control and weight, however, alteration of diet is necessary to maintain the long term effects of FMT.
Wu et al., 2022 [19]	Observational Study	Asses efficacy of fecal microbiota transplantation (FMT) in glycemic control	195 participants - 20 had high blood glucose and 175 normal	FBG, HbA1c, lipid profile, blood pressure (BP), fasting Insulin, HOMA-IR, body mass index (BMI) after 1 month, 2 months and 6 months.	Washed microbiota transplantation (WMT) had a positive effect on glucose profile, BP, and lipid profile in the high blood glucose group. BMI decreased but not significantly.	WMT can provide a new approach in management of hyperglycaemia.
Qiu et al., 2023 [21]	Meta-analysis	Effect of fecal microbiota transplantation (FMT) on Metabolic Syndrome	9 studies -303 participants	Fasting blood glucose (FBG), HbA1c, Insulin, lipid profile, weight, BMI	After 6 weeks, FMT had a positive impact on FBG, HbA1c, Insulin levels, and HDL levels. No significant weight loss.	FMT is beneficial as an alternate treatment for metabolic syndrome, however, more research is needed.
Guzzardi et al., 2023 [20]	Narrative Review	Assessment of fecal microbiota transplantation (FMT) in metabolic disorders	Mixed 5 studies - 146	Glucose, Insulin sensitivity, lipid profile	Beneficial effects on glucose homeostasis, insulin sensitivity, lipid profile, and metabolism. No significant weight loss.	FMT studies provide convincing evidence that gut microbiota can directly cause or reverse impaired glucose tolerance and insulin resistance.
Cai et al., 2018 [22]	Case Report	Role of fecal microbiota transplantation (FMT) in T2DM and its complications	1	Fasting blood glucose (FBG), Postprandial glucose, HbA1c, lipid profile, C peptide, BP, BMI	The clinical parameters significantly improved along with diabetic neuropathy as evidenced by an MRI scan.	FMT may be a new therapeutic option for the management of diabetes and its complications. However, further studies to assess long-term outcomes are needed.

Discussion

Mechanism of Action of FMT and Methods of Transplantation Used

As discussed previously, dysbiosis is linked with the development of type 2 diabetes mellitus and its complications. Alteration in gut microbiota can be achieved by diet, probiotics, prebiotics, and synbiotics [23]. In the last few years, FMT has emerged as a novel method of modulating gut microbiota, which involves the transplantation of feces from healthy individuals to patients with various illnesses.

The research articles included in the systematic review used various methods of FMT (Table 8). The majority of the studies used one method of transplantation such as nasoduodenal tube [14], endoscopy [15,17,22], and oral capsules [18]. However, some studies used two methods of FMT: the nasojejunal tube and transendoscopic enteral tube [19], or the combination of oral capsules and nasoduodenal tube [21]. One article used both oral and direct infusions of FMT [20].

**Table 8:** Methods of fecal microbiota transplantation

Author and year of publication	Method of fecal microbiota transplantation	Courses
Kootte et al., 2017 [14]	Infusion through nasoduodenal tube	Twice - first at baseline and 2nd after 6 weeks
Ng et al., 2022 [15]	Infusion via esophageal gastric endoscopy (OGD)	Once
Ding et al., 2022 [17]	Transendoscopic enteral tubing (TET)	Once over 2 days
Su et al., 2022 [18]	Oral capsules	One capsule on days 1, 8, and 15
Wu et al., 2022 [19]	Nasojejunal tube (upper gastrointestinal) and Transendoscopic enteral tube (lower gastrointestinal)	Twice - one course over 3 days
Qiu et al., 2023 [21]	Capsules and nasoduodenal tube	Mixed
Guzzardi et al., 2023 [20]	Oral and direct infusions	Mixed
Cai et al., 2018 [22]	Endoscopy in terminal ileum	Twice - first at baseline and 2nd after 3 months

FMT has an immunomodulatory effect characterized by decreasing IL-6 and TNF, which is involved in insulin resistance in the liver, muscles, and adipocytes [21]. These pro-inflammatory mediators are also involved in islet cell injury. FMT aims to prevent inflammatory damage to Islet cells, thus restoring pancreatic cell function and improving insulin resistance [19]. In addition, FMT also results in microbiota-derived metabolites that are involved in managing hyperglycemia and improving insulin sensitivity [20]. Short-chain fatty acids (SCFA) butyrate, acetate and propionate, branched chain hydroxy acids, branched-chain fatty acids, acylcarnitines, isobutyric and isovaleric acid, amino acids, and bile acids are some of the microbiota-derived metabolites. Increased levels of these metabolites are driven by FMT alteration in gut microbiota, which has a positive impact on glucose profile and insulin sensitivity. In animal studies, certain bacteria such as *Bifidobacterium, Barnesiellaceae,* and *Fusobacterium* improve blood glucose by increasing the secretion of GLP-1 and inhibiting hepatic gluconeogenesis. Some microbiotas such as *Prevotella, Alloprevotella, Clostridium XlVa, Eubacterium,* and *Intestinimonas* produce SCFAs, which improve blood glucose by stimulating insulin signaling pathways and increasing GLP-1 [24]. Another metabolite, such as acetate, indirectly increases insulin secretion by stimulating the vagus nerve [25].

Role of FMT in Controlling Hyperglycemia and Insulin Resistance (Primary Outcome)

Most of the studies included in the systematic showed the positive impact of FMT in controlling hyperglycemia and other diabetes parameters. One NRCT [17], which involved 17 T2DM patients, showed that after 12 weeks of FMT, there was an improvement in fasting blood glucose (FBG), HbA1c, and C- peptide. Another observational study [18], which assessed the efficacy of FMT (at days 20 and 90) by combining it with diet, showed that FBG and HbA1c significantly reduced in both groups (diet only and diet plus FMT) but slightly better in the FMT group. Another observational study [19], studied the effects of washed microbial transplantation in the short-term (one month), medium-term (two months), and long-term (six months). The results revealed that FMT had a decreasing effect on FBG, HbA1c, fasting insulin (FI), and HOMA-IR. However, the levels of FBG and HOMA-IR did not significantly decrease in the long term.

Meta-analysis and narrative review [21,20], which included various studies, showed that FMT can positively affect glucose profile and insulin sensitivity characterized by reduced FBG and HbA1c and increased insulin levels. In addition to that, a case report [22] that determined the role of FMT in a type 2 diabetic patient concluded that the FMT not only reduced the levels of FBG, postprandial glucose, and HbA1c but also increased the levels of fasting and postprandial C-peptide, and also improved the symptoms of diabetic neuropathy.

Not all studies revealed the positive effects of FMT on glucose profile. One RCT [14], which compared the autologous and allogenic FMT, reduced the levels of HbA1c and improved insulin sensitivity after six weeks. However, the overall glucose levels were not significantly reduced. Another RCT [15] compared the FMT alone and then combined it with lifestyle intervention and sham plus lifestyle intervention. This trial concluded that there was no significant change in fasting blood glucose, fasting insulin levels, and HbA1c in all the groups compared with the baseline.

Role of FMT in Controlling Other Parameters (Secondary Outcome)

The studies included in this systematic review also depicted the effect of FMT on other parameters such as weight, BMI, and lipid levels in addition to diabetes profile. Most studies [14,15,17,19-21] reported no significant weight loss or change in the body mass index (BMI). However, one study [18] concluded significant and earlier weight loss (day 20) in type 2 diabetic patients in FMT and specialized diet. In one case report [22], there was a decline in BMI after the second FMT (22.9 at baseline vs 20.2 after three months).

Regarding lipid profile, all studies except one RCT [14] did show improvement in the lipid profile characterized by a decline in the LDL, total cholesterol, and TG and an increase in HDL. One RCT [15] concluded a significant decrease in LDL and total cholesterol after 24 weeks in the FMT plus Lifestyle intervention (LSI) group. Other NRCT only showed [17] improvement in LDL and TG. In the two observational studies [18,19], patients with high blood glucose saw a declining trend in total cholesterol and TG. One meta-analysis [21] article, which included various studies, only saw improvement in HDL levels, and another narrative review [20] concluded a positive effect of FMT on lipid profile. Another case study [22] revealed a significant decline in TG and LDL.

In addition to the weight loss and lipid profile, blood pressure was also assessed in four studies. Three studies [18,19,22] showed a reduction in systolic blood pressure, while one NRCT [17] showed no significant improvement in systolic blood pressure.

Microbiome Involved in Improving Parameters

Of the studies included in the systematic reviews, only four analyzed the microbiome involved in improving hyperglycemia after FMT (Table 9). One RCT [14] showed raised *Akkermansia muciniphila* and *Eubacterium* genus levels, which correlated negatively to hyperglycemia. Another RCT [15], which included FMT only and FMT combined with LSI, showed an increased presence of butyrate-producing microbiomes such as *Faecalibacterium prausnitzii, Collinsella tanakaei, Anaerostipes hadrus*, several *Eubacterium *spp*.,* and *Coprococcus *spp., which could be involved in controlling hyperglycemia and other parameters.

**Table 9:** Changes observed in the intestinal microbiome after fecal microbiota transplantation

Study	Increase	Decrease
Kootte et al., 2017 [14]	Akkermansia muciniphila Eubacterium genus	Roseburia genus
Ng et al., 2022 [15]	*Butryte-producing Faecalibacterium prausnitzii, Collinsella tanakaei, Anaerostipes hadrus, Eubacterium *spp*. *and* Coprococcus *spp.	*Clostridium clostridioforme* and *Fusobacterium ulcerans*
Ding et al., 2022 [17]		*Rikenellaceae* and the genus *Anaerotruncus*
Su et al., 2022 [18]	*Prevotella, Bifidobacterium, Collinsella, Lactobacillus* and *Neisseria*	*Bacteroides, Bilophila, Lachnospira, Odoribacter, Phascolarctobacterium *and* Sutterella, Desulfovibrio, Butyricimonas, Fusobacterium*
Wu et al., 2022 [19]	NA	NA
Qiu et al., 2023 [21]	NA	NA
Guzzardi et al., 2023 [20]	NA	NA
Cai et al., 2018 [22]	NA	NA

The NRCT done by Ding et al. [17] revealed that *Rikenellaceae* and the genus *Anaerotruncus* significantly decreased to healthy levels after FMT in the responder's group. The levels of these microbiomes were initially high pre-FMT. Finally, in one observational study [18], *Bifidobacterium, Lactobacillus, Collinsella*, and *Neisseria* levels increased significantly and were negatively correlated with blood glucose levels. In contrast, *Desulfovibrio, Butyricimonas, Fusobacterium*, and *Odoribacter* levels declined and were positively correlated with blood glucose levels.

Limitations

There were several limitations seen in the studies included in the systematic review. Firstly, there were inadequate RCTs, and the sample size of type 2 diabetic patients was small. Secondly, not all patients that were included in the study were identified as having type 2 diabetes, as some had pre-diabetes. Thirdly, there was also a variation in diet before FMT was performed in the patients, which could have influenced the glucose metabolism and the results. Moreover, some studies did not use all the parameters in assessing the response of insulin and blood glucose to FMT. In addition, the majority of the studies evaluated the effect of FMT for a short period; therefore, we do not know the long-term efficacy of FMT in managing hyperglycemia. Furthermore, there was an attempt to use an image illustrating the pathophysiology of FMT in controlling blood glucose for its potential benefits. However, obtaining permission from the authors proved difficult due to their lack of response. Finally, only four studies performed a specific analysis of the microbiome involved in glucose metabolism and managing hyperglycemia; therefore, we do not know the specific microbiome involved in blood glucose metabolism.

## Conclusions

This systematic review was done to evaluate the efficacy of FMT in the treatment of insulin resistance and controlling hyperglycemia in type 2 diabetic patients. We not only evaluated the role of FMT in controlling blood glucose but also took into account other factors involved in type 2 diabetes, such as BP, lipid profile, and weight, which can broaden the usage of FMT in treating type 2 diabetes. The studies revealed positive effects of FMT in type 2 diabetes, which is characterized by declining levels of blood glucose, HbA1c, and increased levels of Insulin and C peptide. In addition, FMT also had a positive impact in controlling other parameters such as Blood pressure and lipid profile; however, the effect on weight loss is not convincing based on the studies reviewed. Because there were not enough RCTs and sample size was not enough, more studies with large samples of diabetic patients are required before FMT can be used as standard treatment for type 2 diabetic patients.
